# Oral *Trypanosoma cruzi* Acute Infection in Mice Targets Primary Lymphoid Organs and Triggers Extramedullary Hematopoiesis

**DOI:** 10.3389/fcimb.2022.800395

**Published:** 2022-03-24

**Authors:** Alessandro Marins-Dos-Santos, Jackline de Paula Ayres-Silva, Dina Antunes, Carlos José de Carvalho Moreira, Marcelo Pelajo-Machado, David Alfaro, Agustín G. Zapata, Adriana Cesar Bonomo, Wilson Savino, Juliana de Meis, Désio Aurélio Farias-de-Oliveira

**Affiliations:** ^1^ Laboratory on Thymus Research, Oswaldo Cruz Institute, Oswaldo Cruz Foundation, Rio de Janeiro, Brazil; ^2^ Brazilian National Institute of Science and Technology on Neuroimmunomodulation, Oswaldo Cruz Institute, Oswaldo Cruz Foundation, Rio de Janeiro, Brazil; ^3^ Laboratory of Pathology, Oswaldo Cruz Institute, Oswaldo Cruz Foundation, Rio de Janeiro, Brazil; ^4^ Laboratory of Parasitic Diseases, Oswaldo Cruz Institute, Oswaldo Cruz Foundation, Rio de Janeiro, Brazil; ^5^ Department of Cell Biology, Faculty of Biology, Complutense University of Madrid, Madrid, Spain; ^6^ Rio de Janeiro Research Network on Neuroinflammation, Oswaldo Cruz Institute, Oswaldo Cruz Foundation, Rio de Janeiro, Brazil

**Keywords:** *Trypanosoma cruzi*, oral infection, bone marrow, thymus, hematopoiesis, hematopoietic stem cells, extramedullary hematopoiesis, multiparametric flow

## Abstract

During the acute phase of Chagas disease, *Trypanosoma cruzi* circulation through the bloodstream leads to high tissue parasitism in the host. In primary lymphoid organs, progenitor cell reduction paralleled transient immunosuppression. Herein we showed that acute oral infection in mice promotes diffuse parasitism in bone marrow cells at 14 and 21 days post-infection (dpi), with perivascular regions, intravascular regions, and regions near the bone being target sites of parasite replication. Phenotypic analysis of hematopoietic differentiation in the bone marrow of infected mice showed that the cell number in the tissue is decreased (lineage-negative and lineage-positive cells). Interestingly, analysis of hematopoietic branching points showed that hematopoietic stem and progenitor cells (HSPCs) were significantly increased at 14 dpi. In addition, the pool of progenitors with stem plasticity (HSC-MPP3), as well as multipotent progenitors (MPPs) such as MPP4, also showed this pattern of increase. In contrast, subsequent progenitors that arise from MPPs, such as common lymphoid progenitors (CLPs), lymphoid-primed MPPs (LMPPs), and myeloid progenitors, were not enhanced; conversely, all presented numeric decline. Annexin V staining revealed that cell death increase in the initial hematopoietic branching point probably is not linked to CLPs and that myeloid progenitors decreased at 14 and 21 dpi. In parallel, our investigation provided clues that myeloid progenitor decrease could be associated with an atypical expression of Sca-1 in this population leading to a remarkable increase on LSK-like cells at 14 dpi within the HSPC compartment. Finally, these results led us to investigate HSPC presence in the spleen as a phenomenon triggered during emergency hematopoiesis due to mobilization or expansion of these cells in extramedullary sites. Splenocyte analysis showed a progressive increase in HSPCs between 14 and 21 dpi. Altogether, our study shows that the bone marrow is a target tissue in *T. cruzi* orally infected mice, leading to a hematopoietic disturbance with LSK-like cell bias accounting on HSPCs possibly affecting myeloid progenitor numbers. The LMPP and CLP reduction converges with defective thymocyte development. Lastly, it is tempting to speculate that the extramedullary hematopoiesis seen in the spleen is a mechanism involved in the hematological maintenance reported during the acute phase of oral *T. cruzi* infection.

## Introduction

Hematopoietic stem and progenitor cells (HSPCs) are capable of differentiating, sustaining, and replenishing the hematopoietic and immune system cell pools. The widely accepted concept of hierarchical hematopoietic differentiation reports uncommitted hematopoietic stem cells (HSCs) in the top of the hematopoietic branching point (Kondo et al., 1997; Akashi et al., 2000; Nakorn et al., 2003). These cells are recognized as being negative for lineage markers and present the stem phenotype Lin^−^Sca-1^+^c-Kit^+^ (LSK cells). Yet the LSK population is heterogeneous, and the positive modulation of the protein FLT3 characterizes multipotent progenitors (MPPs) harboring the phenotype Lin^−^Sca-1^+^c-Kit^+^FLT3^+^ (Adolfsson et al., 2005). The FLT3 protein is mostly found on the surface of progenitors with robust multipotency and the ability to differentiate into lymphoid progenitors. MPPs have been shown to be richer in FLT3^+^ cells as compared to the HSC population (Mooney et al., 2017). One broadly applicable scheme separates the most quiescent HSCs (CD34^−^/Flk2^−^/CD150^+^/CD48^−^ LSK) from the more metabolically active MPP1 (CD34^+^/Flk2^−^/CD150^+^/CD48^−^ LSK) and subdivides MPPs into three further distinct subsets: MPP2 (Flk2^−^/CD150^+^/CD48^+^ LSK), MPP3 (Flk2^−^/CD150^−^/CD48^+^ LSK), and MPP4 (Flk2^+^/CD150^−^/CD48^+/−^ LSK) (Pietras et al., 2015).

These MPPs maintain the ability to differentiate into all blood cell types but do not self-renew as HSC (Adolfsson et al., 2005; Boyer et al., 2011). Further commitment to the myeloid lineage is defined by the negative loss of Sca-1, turning into Sca-1^−^Lin^−^c-Kit^+^ population (Pu et al., 2016), while common lymphoid progenitors (CLPs) are characterized by the Lin^−^Sca-1^+^c-Kit^+^FLT3^+^CD127^+^ phenotype (Adolfsson et al., 2005).

The progenitor cell differentiation is regulated in the inter-sinusoidal spaces of the bone cavity that extends from the endosteum to the endothelial cell membrane of the sinusoids (Osawa et al., 1996). In this sense, microenvironmental cells foment instructive signals, guiding the HSPCs’ fate in the bone marrow niche (Kondo, 2010; Brown et al., 2015; Cordeiro Gomes et al., 2016). Actually, the bone marrow microenvironment operates as an absolute requirement for the maintenance of HSCs and their future fate (Allen and Dexter, 1984). In fact, HSPCs are influenced by different cellular, extracellular, and soluble mediators. Among them, the crosstalk with the immune system has been pointed as being central in hematopoietic regulation (Monteiro et al., 2005; Monteiro and Bonomo, 2005; Mirantes et al., 2014; Bonomo et al., 2016).

Like mature immune cells, HSCs can be activated directly by pathogen recognition receptors (PRRs) *via* Toll-like receptors (TLRs), or proinflammatory cytokine signals based on IL-6, TNF-α, and IFN-γ overproduction (Takizawa et al., 2017).

Following oral *Trypanosoma cruzi* infection, the immune response drives a powerful proinflammatory scenario, comprising elevated levels of plasma IFN-γ and TNF-α, favoring control of the parasite burden (Barreto-de-Albuquerque et al., 2015). Recently, the systemic influence of the *T. cruzi* infection has been reported leading to changes in bone marrow homeostasis causing anemia, thrombocytopenia, leucopenia, and bone marrow hypoplasia. Moreover, transference of bone marrow cells from infected to irradiated mice failed to replenish host hematopoiesis (Marcondes et al., 2000).

The *T. cruzi*-induced functional unbalance of the hematopoietic bone marrow is accompanied by a deficiency in IL-7 production by microenvironmental cells, resulting in impairment of pro- and pre-B-cell development (Muller et al., 2018). Furthermore, thymic atrophy occurring in mice following classic i.p. acute *T. cruzi* infection apparently involves a decay in CLP production in the bone marrow with parallel reduction in late differentiation stages of immature CD4^−^CD8^−^ (DN) thymocytes (Carbajosa et al., 2017). These findings suggest that *T. cruzi* infection elicits bone marrow disturbance, leading to a reduction in precursor cells engaged in the replenishment of the B-cell and T-cell pools. Both populations are pointed as sources of key cytokines and effector activities that contribute to parasite replication control (Lenardo, 1991; Lopes and DosReis, 1996).

Albeit evidence demonstrates that *T. cruzi* infection induces disturbances in committed progenitors, the impact on progenitors with stem and multipotency as HSCs and MPPs is unknown. Additionally, most studies rely on intraperitoneal experimental infection, which is an unnatural route of transmission and critically generates a different feature on host immune response and disease outcome (Barreto-de-Albuquerque et al., 2015; Silva-Dos-Santos et al., 2017). In the last 10 years, oral *T. cruzi* infection has accounted for more than half of the cases of acute Chagas disease in the Brazilian Amazon. Oral infection leads to more severe disease than vectorial transmitted disease, with an 8%–35% mortality rate in comparison with the classical vectorial transmission death rates of less than 10% (Rassi et al., 2010; Filigheddu et al., 2017).

Recently, we established a model of experimental oral infection, which recalls the human disease (Barreto-de-Albuquerque et al., 2015; Silva-Dos-Santos et al., 2017). Using this model, we investigated the presence of the parasite in the bone marrow environment and its impact on the balance of progenitors in each hematopoietic branching point, extending to changes in the thymus.

## Methods

### Mice and Ethics Statement

BALB/c male mice aged 6–8 weeks were obtained from the Science and Technology Institute for Biomodels, Oswaldo Cruz Foundation (Rio de Janeiro, Brazil). Mice were maintained under specific pathogen-free conditions and were used according to protocols approved by the Institutional Ethics Committee for Animal Research of the Oswaldo Cruz Foundation (CEUA-Fiocruz, License: L-028/2016). The experiments were performed in strict accordance with the recommendations in the Guide for the Care and Use of Laboratory Animals of the Brazilian National Council of Animal Experimentation and the Federal Law 11.794 (October 2008).

### Parasite and Oral Infection

*T. cruzi* freshly obtained from excreta of infected *Triatoma infestans* in the 5th stage of development, or trypomastigote forms obtained from infected VERO cell line supernatants, were provided for infection. Mice were starved 4 h before and 15 min after oral parasite inoculation; 30 μl of the *triatomine* excreta or Roswell Park Memorial Institute (RPMI) medium containing 5 × 10^4^ parasites of the *Tulahuen* strain DTU TcVI of *T. cruzi* were released into mouse oral cavity. Parasite quantification in the bloodstream was performed through the Pizzi–Brenner method between 7 and 50 dpi.

### Sampling and Histological Processing

Tibias, femurs, and humeri were obtained and fixed for 48 h in Carson’s Millonig formalin at room temperature, and mineralized bone decalcification was made through EDTA protocol (Carson et al., 1973). Samples were processed in a Shandon Citadel 2000 tissue processor (Thermo, Waltham, MA, USA) according to standard histological techniques for paraffin embedding. Paraffin sections with a thickness of 5 μm were obtained in a rotary microtome (Microm HM-325 or Leica RT2125). These sections were de-waxed in xylol and hydrated in decreasing ethanol concentrations until being washed in distilled water to prepare for staining with H&E and Sirius red. Slides with sections from tibias, femurs, and humeri were analyzed in an Axiovert 200M microscope and the images were acquired with an AxioCam HRc color camera (Carl Zeiss, Oberkochen, Germany).

### Immunofluorescence

Paraffin sections with a thickness of 5 μm were de-waxed in xylol, hydrated in decreasing ethanol concentrations, and washed in distilled water to prepare for heat-induced epitope retrieval, using acidic buffer (citrate pH 6.0) at a Pascal chamber (DAKO, Carpinteria, CA, USA) for 3 min. After being cooled at room temperature for 10 min, slides were washed in distilled water and then with phosphate-buffered saline (PBS) before being incubated overnight at 4°C with a mouse anti-*T. cruzi* primary antibody (1:400) obtained from mice infected with *Tulahuen* strain. After 1 day, slides were washed 3 times in PBS, incubated for 1 h at 37°C with goat anti-mouse AlexaFluor 488 secondary antibody (A-11001, Thermo Fisher, Waltham, MA, USA), washed 3 times with PBS, counterstained during 10 min with 1:15,000 DAPI (4′,6-diamidino-2-phenylindole, D1306, Invitrogen, Carlsbad, CA, USA), diluted in PBS, and mounted with ProLong gold (P36934, Life Technologies, Carlsbad, CA, USA). Histological sections were acquired using an LSM 710 confocal laser microscopy (Zeiss, Germany) equipped with AxioObserver Z2.

### Flow Cytometry

Bone marrow cells were flushed from tibias or femurs and humeri in RPMI 10% fetal calf serum (Cultilab, São Paulo, Brazil), homogenized, and eluted through a Falcon 70-μm cell strainer (Corning, New York, USA). Red blood cell lysis buffer (Merck, Darmstadt, Germany) was used in the isolation of HSPCs, and bone marrow cells were counted using a Neubauer chamber.

The HSPC phenotypes were characterized as HSCs Lin^−^SCA^−^1^+^cKit^+^FLT3^−^, MPP as Lin^−^SCA-1^+^cKit^+^FLT3^+^, CMP as Lin^−^SCA^−^1-cKit^+^FLT3^−^, lymphoid-primed MPP (LMPP) as Lin^−^SCA-1^+^cKit^+^FLT3^+^CD127^−^, and CLP as Lin^−^SCA-1^+^cKit^+^FLT3^+^CD127^+^. The gating strategies were performed using Boolean analyses (**Supplementary Figure 1**). Lin^+^ cells were further evaluated by immunostaining for the expression of CD3ϵ, CD11b, CD45R, B220, TER-119, Ly-6G, and Ly-6C (**Supplementary Table 1**). APC-coupled mouse lineage isotype control cocktail contained equivalent concentrations of isotype-matched negative-control immunoglobulins. Annexin staining was performed as recommended on annexin V staining protocol (Thermo Fisher Scientific, Waltham, MA, USA) and used to identify dead cells as shown on the annexin histogram in the middle of the panel (**Supplementary Figure 1**).

A total of 100,000–200,000 events of freshly isolated samples were collected within the morphological gate using CytoFLEX high-performance flow cytometer (Beckman Coulter, Indianapolis, IN, USA). HSPC analysis was achieved after gating in singlets and by the limit of autofluorescence found in isotype control or unlabeled cells. Fluorescence spread, compensation test, and gate adjustment were done using the Fluorescence Minus One (FMO) method.

Thymuses were removed and minced, and thymocytes were washed and incubated in PBS containing 3% fetal calf serum (Gibco, California, USA). For immunostaining, 1 × 10^6^ thymocytes were incubated with a given mix of specific fluorochrome-conjugated monoclonal antibodies for 30 min at 4°C in the dark. The mix comprised antibodies with the following specificity: Lin cocktail, CD4, CD8, CD44, CD25, and CD16/32 (for Fc receptor blockage). Negative controls were defined from fluorochrome-conjugated antibody isotype controls (Becton Dickinson reagents, Becton Dickinson, Franklin Lakes, NJ, USA).

HSPCs in the spleen were investigated by labeling splenocytes with Lin, Sca-1, c-Kit, and CD16/32 (to block Fc receptors).

In all cases, flow cytometry acquisition was performed through the CytoFLEX high-performance flow cytometer (Beckman Coulter, Indianapolis, USA) and analyzed using the Kaluza software.

### Statistical Analyses

All statistical analyses were done using the GraphPad Prism 6 software (GraphPad Inc.). Data were subjected to Shapiro’s normality test to determine whether they were sampled from a Gaussian distribution. The Kruskal–Wallis with Dunn’s multiple comparison test or ANOVA with Tukey’s multiple comparison test was applied for samples that, respectively, deviated or assumed a Gaussian distribution; outliers were calculated by Grubbs’ test. When *p* < 0.05, differences between groups were considered statistically significant.

## Results

### Bone Marrow Is a Target Organ in Orally Induced *Trypanosoma cruzi* Acute Infection

During the acute phase of oral *T. cruzi* infection, trypomastigote forms disseminate through the bloodstream (**Supplementary Figure 2**) and infect different organs as previously reported (Silva-Dos-Santos et al., 2017). Although it was shown that the spleen, liver, and brain as well as different segments of the digestive tract were infected, the bone marrow had never been evaluated after oral infection.

**Figure 1:**
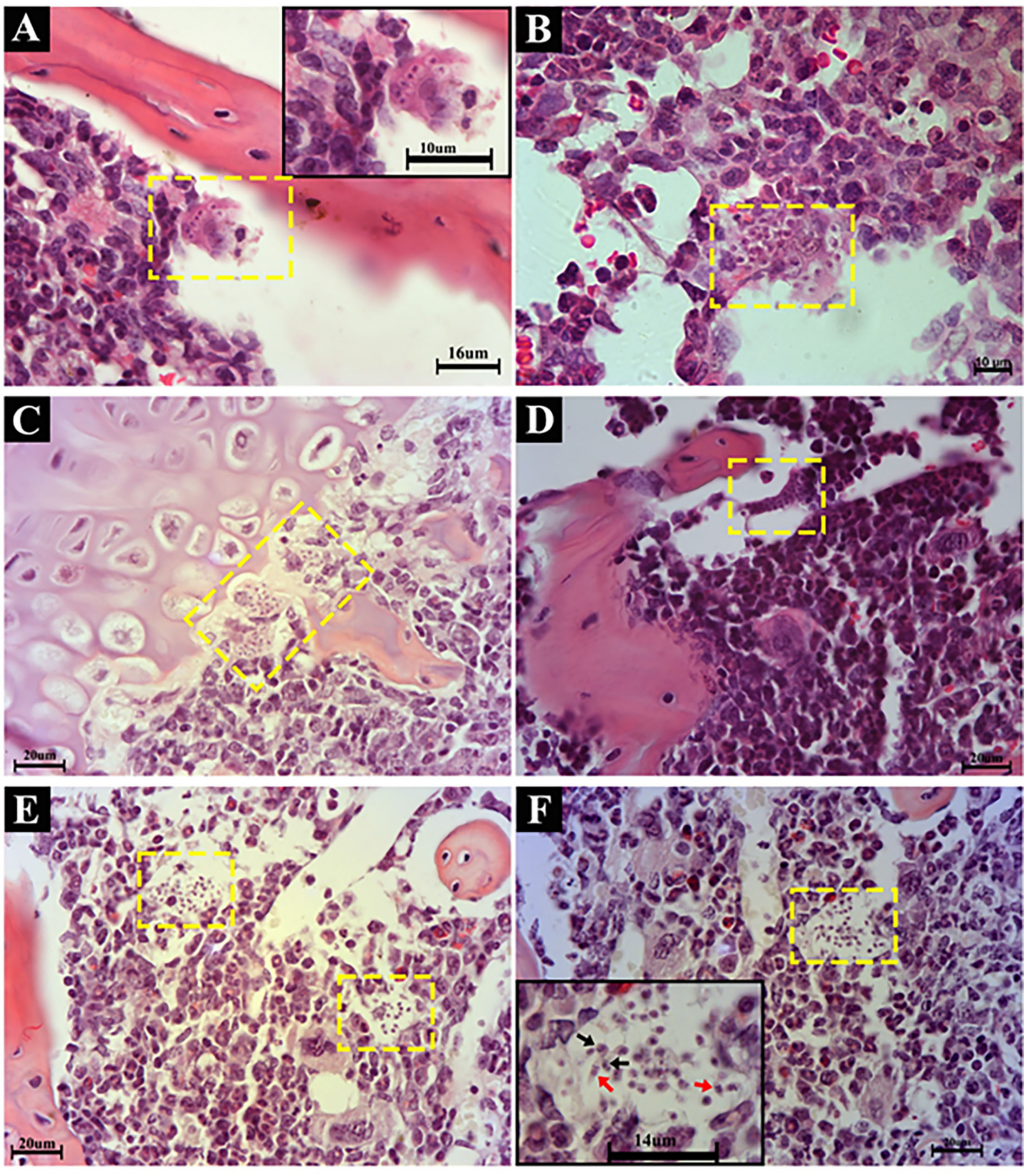
Oral *Trypanosoma cruzi* acute infection induces parasitism in different sites of the bone marrow. **(A**, **B)** The microarchitecture of the mouse femur at 14 and 21 dp, showing amastigote forms of the parasite in stromal cells (yellow dashed rectangles) near the bone spicula. The Insert shows an infected cell in higher magnification. **(C)** The histological profile of the femur from one infected mouse at 14 dpi showing parasitism in the cartilage–bone intersection (yellow dashed rectangle). **(D)** Histology of the humerus of an infected mouse at 21 dpi, in which amastigotes in the perivascular space and near bone spicule can be seen. **(E)** A histological section of the humerus from an infected mouse at 21 dpi showing amastigote forms in bone marrow stromal cells. **(F)** Histology of the humerus from an infected mouse at 21 dpi, revealed in the inset as elongated forms with typical kinetoplasts (red arrows) and amastigote forms of the parasite within bone marrow microenvironmental cells (black arrows). Tissue sections were stained by H&E. The data are representative of 5 mice per group, 14 and 21 dpi. Bars show the magnification of each micrograph in each panel, as well as insets.

To investigate parasitism in the bone marrow, corresponding tissue sections were evaluated at 7, 14, and 21 dpi by H&E staining. Parasites were not detected at 7 dpi (data not shown), although nests of *T. cruzi* amastigotes were clearly seen in cells close to the bone matrix at both 14 and 21 dpi (**Figures 1A, B**). The infection also comprised cells at the cartilage intersection with the nascent osseous tissue and perivascular region (**Figures 1C, D**). The presence of both amastigote and trypomastigote forms of the parasite suggests that the bone marrow might be an active site of replication and release of the parasite at 21 dpi (**Figures 1E, F**), as previously suggested (Morocoima et al., 2006). Immunofluorescence analysis performed at 14 and 21 dpi confirmed parasitism in different regions of the bone marrow (**Supplementary Figure 3**).

### Hematopoietic Progenitors Are Affected After Oral *Trypanosoma cruzi* Infection

**Figure 2:**
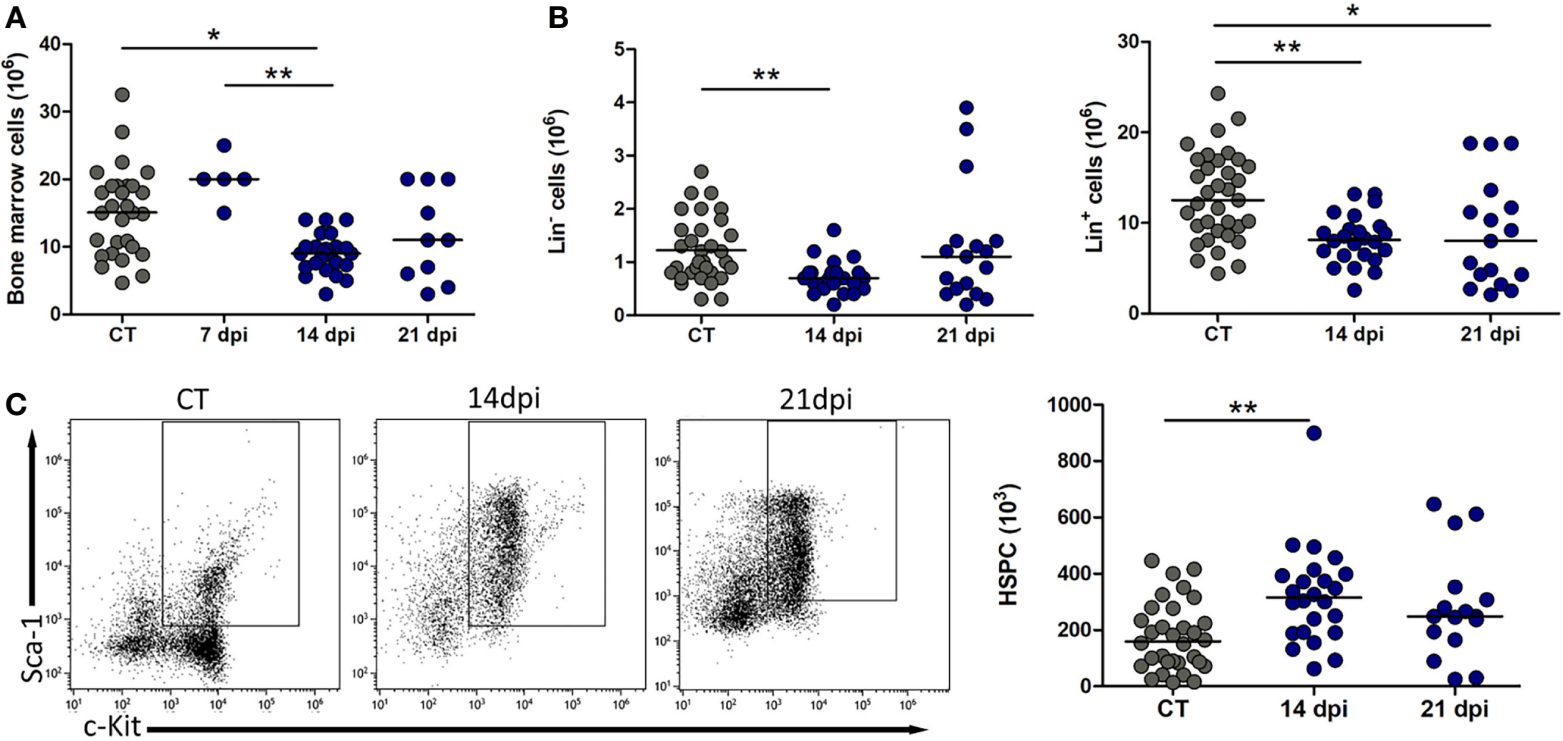
Oral *Trypanosoma cruzi* acute infection alters bone marrow hematopoiesis. **(A, B)** A transient, yet significant, reduction in total cell numbers and the immature Lin^−^ components. Total numbers of Lin^+^ cells are also down at 14 dpi, but differently, remained lower than controls even at 21 dpi, as seen in Panel **(B, C)** Representative dot plots of Sca-1 *versus* c-kit expression gated in Lin^−^ cells and HSPC absolute cell numbers, with phenotype Lin^−^SCA-1^+^c-Kit^+^. Bars indicate the medians in each group; circles depict individual mice. The data comprised results from 5 infected animals at 7 dpi, or pooled from 5 independent experiments with 3–7 animals per analysis at 14 dpi, or pooled from 5 independent experiments with 2–7 animals per analysis at 21 dpi. All control animals were grouped in each setting. Control *versus* infected groups compared through a non-parametric Kruskal–Wallis ANOVA test with Dunn’s multiple comparison test. *p < 0.05, **p < 0.001.

Since the bone cavity harbors hematopoiesis, it was reasonable to ask if the presence of *T. cruzi* nests within the bone/blood interface was affecting hematopoiesis. The total numbers of bone marrow cells were decreased at 14 dpi and recovered by 21 dpi (**Figure 2A**). Following analysis of Lin^−^ and Lin^+^ populations, we found that both were diminished at day 14, suggesting smaller numbers of uncommitted and committed progenitors (**Figure 2B**). Such a decrease was restored at 21 dpi only for the more immature Lin^−^ progenitors, with cell numbers being similar to those found in non-infected mice. Differently, the more mature Lin^+^ subpopulation remained low (**Figure 2B**), indicating an arrest at later points of differentiation. Moreover, the quantification of the HSPC population (Lin^−^c-kit^+^Sca-1^+^) shows increased numbers of immature progenitors at 14 dpi within Lin^−^ population, suggesting that hematopoiesis could be demanded (**Figure 2C**).

**Figure 3:**
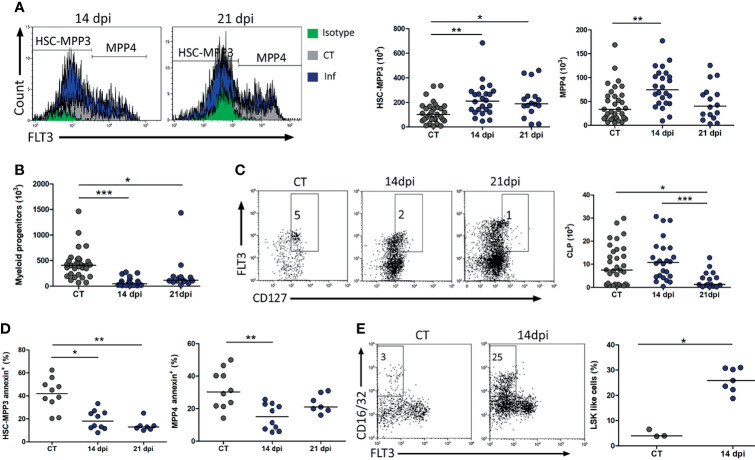
Augmentation of HSCs and MPPs in forward oral *Trypanosoma cruzi* infection without parallel CLPs and myeloid progenitor expansion in bone marrow occur concomitantly with LSK-like cells increase. **(A)** Representative histogram of HSCs FLT3^−^ and MPPs FLT3^+^ gated within Lin^−^SCA-1^+^c-kit^+^CD127^−^ cells and absolute cell number of HSCs (Lin^−^SCA-1^+^c-kit^+^FLT3^−^CD127^−^) and MPPs (Lin^−^SCA-1^+^c-kit^+^FLT3^+^CD127^−^) at 14 and 21 dpi. **(B)** Absolute cell number of myeloid progenitors at 14 and 21 dpi. **(C)** Representative dot plot of CLPs FLT3 versus CD127 gated within Lin^−^SCA-1^+^c-kit^+^ cells and absolute cell number of CLPs (Lin^−^SCA-1^+^c-kit^+^ FLT3^+^CD127^+^) at 14 and 21 dpi. **(D)** Annexin-defined cell death rate on HSCs and MPPs at 14 and 21 dpi. **(E)** Representative dot plot of CD16/32^+^ gated within Lin^−^SCA-1^+^c-kit^+^ cells and absolute cell number of LSK-like cells (Lin^−^SCA-1^+^c-kit^+^CD16/32^+^) at 14 dpi. Representative dot plot of increased SCA-1 expression gated within Lin^−^ cells shown previously in dot plot in **Figure 2C**. Bars indicate median; circle depicts individual mice. The data are representative of pooled results from 5 independent experiments with 2–7 animals per analysis at 14 dpi and 4 independent experiments with 2–5 animals per analysis at 21 dpi **(A–C)**. Statistical analyses of cell death were pooled from 2 independent experiments with 3–7 animals per analysis at 14 dpi and 1 independent experiment with 7 animals at 21 dpi. All CT animals were grouped in the same set. Non-parametric, Kruskal–Wallis ANOVA test with Dunn’s multiple comparison test [**(A–C)** and HSCannexin] and one-way analysis of variance ANOVA test with Tukey’s multiple comparison test (MPP annexin). *p < 0.05, **p < 0.001, ***p < 0.0001. HSCs, hematopoietic stem cells; MPPs, progenitors with multipotency; CLPs, common lymphoid progenitors; CT, control.

We further investigated the maintenance of the different progenitors throughout the hematopoietic hierarchy, aiming at defining the point of possible unbalance. We designed a multiparametric flow cytometry evaluation detailing the characterization of HSCs to MPP3 (Lin^−^c-kit^+^Sca-1^+^FLT3^−^CD127^−^) and MPP4 (Lin^−^c-kit^+^Sca-1^+^FLT3^+^CD127^−^). MPPs pursue stem plasticity to differentiation and replenishment of committed myeloid progenitors as CMPs–GMPs (Lin^−^c-kit^+^Sca-1^−^CD16/32^+^) and CLPs (Lin^−^c-kit^+^Sca-1^+^FLT3^+^CD127^+^). Our results revealed that HSPC numbers were sustained mainly by the increase of the HSC-MPP3 pool in both 14 and 21 dpi, as well as MPP4 at 14 dpi (**Figure 3A**). By contrast, lineage-committed progenitors as myeloid showed a remarkable decline at 14 and 21 dpi, while CLPs were unchanged at 14 dpi but significantly decreased at 21 dpi (**Figures 3B, C**).

### Cell Death Does Not Account for the Diminished Numbers of Hematopoietic Progenitors

We addressed whether annexin V-positive HSC-MPP3 pool and MPP4 could account for the smaller numbers of lineage-committed progenitors following oral *T. cruzi* acute infection. The lower frequency of annexin V on HSC-MPP3 pool and MPP4 population (**Figure 3D**) makes it tempting to speculate that a possible cell death increase in this initial hematopoietic branching point does not seem to be associated with the minor output of the myeloid and CLPs forward hematopoietic demands at 14 dpi and CLPs at 21 dpi.

Subsequently, we also investigated whether oral *T. cruzi* infection becomes altered on the hematopoietic program, eliciting a phenotypic bias. Accordingly, we found an increase in Sca-1 expression on HSPCs. Previous work reported the LSK-like cells from CMPs-GMPs CD16/32^+^ as bearing an atypical expression of Sca-1 (Yang et al., 2017). The presence of this population was significantly expanded in frequency at 14 dpi within the HSPC compartment (**Figure 3E**).

### Bone Marrow Lymphoid-Primed Progenitors With Multipotency and Intrathymic T-Cell Development Are Also Impaired in *Trypanosoma cruzi* Orally Infected Mice

**Figure 4:**
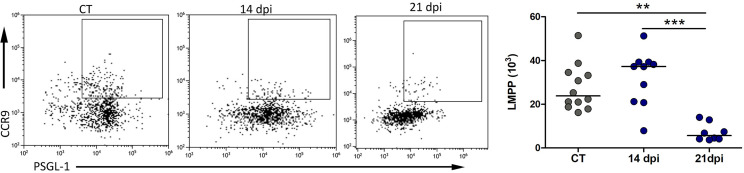
Reduced numbers of downstream LMPPs in the bone marrow of *Trypanosoma cruzi* orally infected mice. Representative dot plots of LMPP (Lin^−^Sca-1^+^c-kit^+^FLT3^+^PSGL-1^+^CCR9^+^) and absolute cell number along with days post-infection. Bars indicate the medians in each group; circles depict individual animals. The data comprised results from 3–12 mice at 14 and 21 dpi. Control animals were grouped in each setting. Control *versus* infected groups compared through non-parametric, Kruskal–Wallis ANOVA test with Dunn’s multiple comparison test. **p < 0.001, ***p < 0.0001. LMPPs, lymphoid-primed progenitors with multipotency.

The immune cell replenishment and lymphoid organ settling by precursors are dependent on a dynamic process of polarization. For T cells, the LMPP with high migratory potential to the thymus comprises a wide PSGL-1^+^CCR9^+^ subset in MPPs and CLPs. We found a significant decrease in the number LMPPs at 21 dpi within the bone marrow (**Figure 4**).

**Figure 5:**
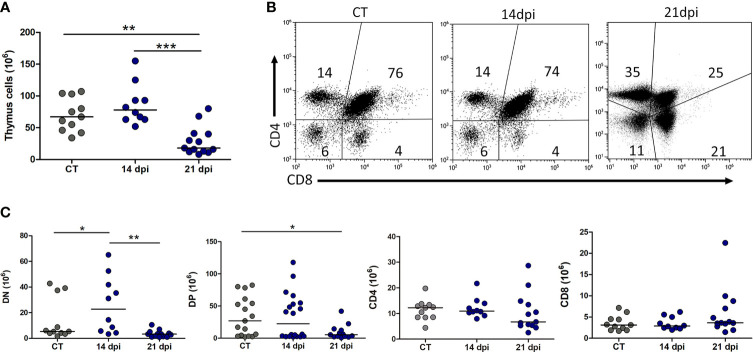
Progressive thymic atrophy with CD4^+^CD8^+^ thymocyte depletion in mice subjected to *Trypanosoma cruzi* oral acute infection. **(A)** Progressive decrease in total thymocyte numbers. **(B)** CD4/CD8-defined cytofluorometric dot plots revealing progressive loss in the relative numbers of CD4^+^CD8^+^ double-positive thymocyte subset. The data are representative of 2 experiments with 3–5 animals per group at 14 dpi and 2 independent experiments with 2–5 animals per analysis at 21 dpi. **(C)** Absolute numbers of each CD4/CD8 cell. Statistical differences were ascertained with the non-parametric, Kruskal–Wallis ANOVA test with Dunn’s multiple comparison test. *p < 0.05, **p < 0.001, ***p < 0.0001.

Since a decrease in the number of lymphoid progenitors could influence thymocyte differentiation, it seemed worthy to evaluate the thymus following acute oral infection. In fact, the total numbers of thymocytes diminished at day 21, reaching all CD4/CD8-defined subpopulations (**Figure 5**).

**Figure 6:**
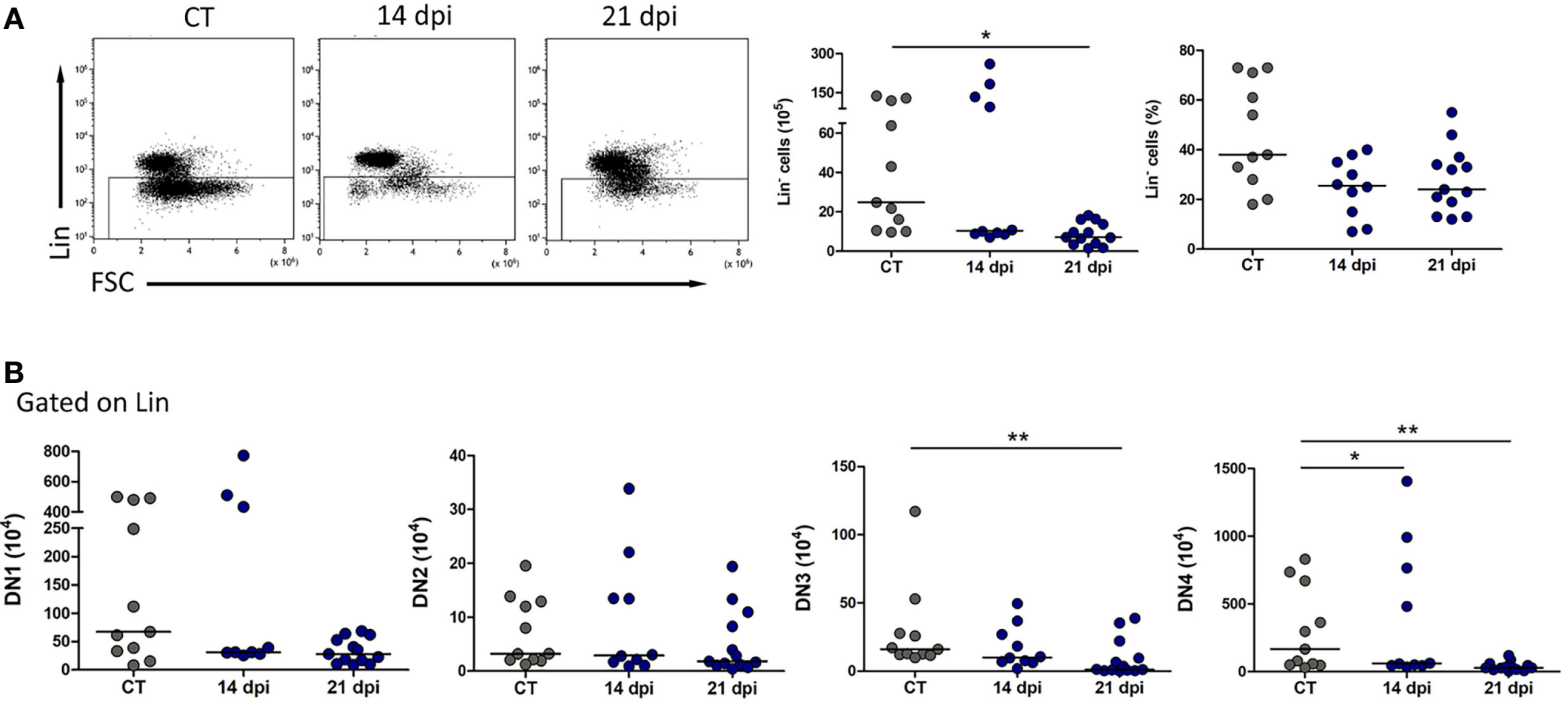
Decrease in the relative numbers of CD4^−^CD8^−^ immature thymocyte subsets following *Trypanosoma cruzi* acute oral infection. **(A)** Decreased absolute (but not relative) numbers of Lin^−^ population. **(B)** Within the Lin^−^ compartment, DN thymocytes were followed by immunostaining with CD25 and CD44. In late oral *T. cruzi* infection, the absolute numbers of virtually all DN subsets (except for DN2 cells) were significantly decreased in late days post-infection. The data are representative of 2 experiments with 3–5 animals per group at 14 and 21 dpi. Statistical differences were ascertained with the non-parametric, Kruskal–Wallis ANOVA test with Dunn’s multiple comparison test. *p < 0.05, **p < 0.001.

As previously shown for i.p. *T. cruzi* infection, the main thymocyte population affected is the DP, usually targeted by the glucocorticoid-related stress response (Perez et al., 2007; Savino, 2017). However, lack of progenitors could also contribute to the low thymocyte number, and in this case, it should be reflected in the number of DN population. Total numbers of DN thymocytes increase at day 14, before thymic atrophy, and return to control levels by day 21, suggesting demand for progenitors, coming from the bone marrow (**Figure 5C**). Paradoxically, the very immature Lin^−^ population remains with decreasing tendency at 14 and 21 dpi (**Figure 6A**).

We then checked the DN stages defined by the expression of CD25 and CD44 within the Lin^−^ population. In late oral *T. cruzi* infection (21 dpi), when thymic atrophy is evident, the absolute numbers of virtually all DN subsets (except for DN2 cells) tend to diminish (**Figure 6B**). We found a decrease in the absolute numbers of DN Lin^−^ population (**Figure 6A**), suggesting that either smaller numbers of immature migrants are arriving from the bone marrow or they are further differentiating into more mature subsets. Both possibilities are plausible. Of note is the variability in the results obtained in individual mice by 14 dpi.

### *Trypanosoma cruzi* Oral Infection Triggers Splenic Hematopoiesis

The results observed in the hematopoietic bone marrow, regarding the increase in HSC-MPP3 pool and MPP4, suggest that hematopoiesis is being demanded. We thought that if the hematopoietic request is indeed the case, splenic hematopoiesis could be present (Oda et al., 2018).

**Figure 7:**
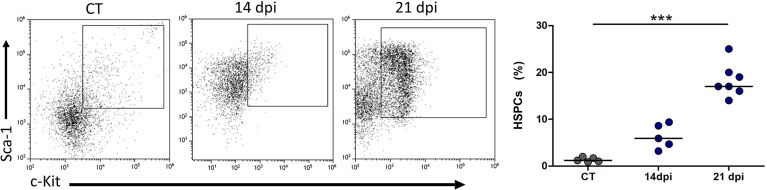
Extramedullary hematopoiesis during oral *Trypanosoma cruzi* infection. Representative dot plots of Sca-1 *versus* c-kit expression gated in the Lin^−^ compartment and relative number of HSPCs (Lin^−^Sca-1^+^c-Kit^+^) in the spleen. Bars indicate median; circle depicts individual mice in infected (14 and 21 dpi) and control (CT) groups. The data are representatives of 1 experiment with 5–7 animals per group. Statistical differences were ascertained with the non-parametric Kruskal–Wallis ANOVA test with Dunn’s multiple comparison test. ***p < 0.0001. US, stained cells.

To tackle this issue, we performed a histologic evaluation of the spleen to check for active hematopoiesis. At 14 and 21 dpi, we found enlarged spleens, with the presence of megakaryocytes and myelocytes. Moreover, a hypertrophic red pulp along with an atrophic white pulp due to follicle coalescence was observed (**Supplementary Figure 4**). Additionally, we observed a massive increase of HSPC frequency in the spleen at 21 dpi when compared with control animals (**Figure 7**).

Considering our previous results demonstrating partial hematological changes in the complete blood cell analysis (Antunes et al., 2019), we speculate that HSPC expansion in the spleen is in line with the emergency hematopoiesis triggered by oral *T. cruzi* infection.

## Discussion

Clinical studies have demonstrated that outbreaks of acute Chagas disease caused by consumption of food contaminated with *T. cruzi* might induce severe cases with relatively high lethality (Alarcon de Noya et al., 2010; Shikanai-Yasuda and Carvalho, 2012). In the current study, we documented for the first time an experimental model using the oral route of acute *T. cruzi* infection that leads to parasitism in the bone marrow, compromises thymocyte development, and augments HSPC number in the spleen.

Previously, we used a model of oral infection with luciferase transgenic *T. cruzi* and confirmed by real-time PCR that the parasite disseminates systemically. Sites of infection in the nasomaxillary region and the whole digestive system plus the mesentery, liver, spleen, heart, brain, and testicles were shown at later moments of the acute phase (Silva-Dos-Santos et al., 2017).

In the present work, we found active sites of infection and parasite replication in the bone marrow, where cells with eosinophilic cytoplasm are close to the bone matrix and perivascular region and present large amastigote nests. This is in line with a previous report showing chondrocytes and bone marrow macrophages infected after i.p. injection of the parasite (Morocoima et al., 2006).

Our histopathological observation was confirmed by immunofluorescence analysis revealing diffuse parasitism in the bone marrow at 14 and 21 dpi. Coincidentally, both time points paralleled elevated levels of parasitemia when compared with 7 dpi, when parasitism was not found.

The presence of *T. cruzi* in the bone marrow in human Chagas disease is unknown. Albeit information concerning hematological disorders during the acute phase of human oral Chagas disease is scarce, individuals had higher plasma levels of activated protein C and lower levels of factor VII of the coagulation cascade among the clinical manifestations. Events such as anemia or blood cell count abnormality were not mentioned (de Goes Costa et al., 2017; Santos et al., 2020).

The activation of an effector program is critical for the control of the parasite burden during the acute phase of Chagas disease. Albeit few studies are available in the literature covering a qualitative analysis of human immune response during acute *T. cruzi* infection, T-cell responses and antibody production, as well as activation of innate mechanisms, seem to be important for parasite control during the acute phase (Campos and Gazzinelli, 2004; Krettli, 2009; Andrade et al., 2014). It is noteworthy to mention that parasite control during the acute phase is highly efficient, given that parasitemia is undetectable as patients enter the chronic phase. Studies in murine models of *T. cruzi* infection have shown that a robust inflammatory response triggered in the acute phase, with cytokines such as IFN-γ and TNF-α, was considered pivotal (Silva et al., 1995; Marins-Dos-Santos et al., 2020). This assumption is supported by the findings that IFN-γ and TNF-α knockout animals are highly susceptible to *T. cruzi* infection, as are nitric oxide-deficient mice (Romanha et al., 2002). During the acute phase of oral *T. cruzi* infection, high plasma levels of IFN-γ and TNF-α were also reported (Barreto-de-Albuquerque et al., 2015; Antunes et al., 2019). Similar to TLRs, inflammatory signaling molecules, including IFN-γ and TNF-α, induce HSC response (Baldridge et al., 2010).

Thus, we raised the hypothesis that transversally, this infection scenario affects bone marrow function, reducing the ability to sustain bone marrow cellularity at 14 dpi. Based on earlier investigations, *T. cruzi* infection through the subcutaneous and intraperitoneal routes induces a decrease in the numbers of myeloblasts, monoblasts, megakaryoblasts, erythroblasts, pro- and pre-B cells, and CLPs, thus contributing to a reduced potential of immune cell replenishment (Marcondes et al., 2000; Carbajosa et al., 2017; Muller et al., 2018).

The hematopoietic function providing committed progenitor maintenance is dependent on progenitors with stem or multipotency ability (Nakorn et al., 2003; Akashi et al., 2005; Luc et al., 2008). In line with this concept, we documented herein a decrease in Lin^−^ population. Interestingly, the number of HSPCs was transiently increased at 14 dpi, keeping similar numbers when compared to control mice until our last point of investigation at 21 dpi. However, the increased number of HSC-MPP3 pool and MPP4 does not seem to contribute sufficiently to repopulate myeloid progenitors and CLPs, at least inside the bone marrow. In this regard, a detailed analysis separating HSCs and MPPs based on CD34, CD150, and CD48 expression, besides using FLT3, could enrich our data regarding the disturbance of quiescent stem cells and MPPs (Oguro et al., 2013; Cabezas-Wallscheid et al., 2014; Mooney et al., 2017).

Expansion of the myeloid compartment is often mediated by proinflammatory cytokines, microbial products, or hematopoietic emergencies (Baldridge et al., 2010; Belyaev et al., 2013). Emergency GMPs are associated with high proliferative potential, thus resulting in the generation of a larger progeny in the periphery (Buechler et al., 2013). Conversely, we found decreased myeloid progenitors in the bone marrow together with a concomitant increase of LSK-like cells.

In addition, the LMPPs in the bone marrow, which manifest a high potential to colonize the thymus, are reduced. This result agrees with recent findings, in which a significant decrease in bone marrow lymphoid progenitors during acute *T. cruzi* infection occurred concomitantly with thymus atrophy and alterations in immature thymocytes (Carbajosa et al., 2017).

Activated proliferating HSPCs and myeloid progenitor cells leave the bone marrow and migrate to the spleen, liver, and other inflamed target organs (Griseri et al., 2012; Burberry et al., 2014; Haas et al., 2015). Additionally, infection with *Ehrlichia muris* and *Anaplasma phagocytophilum* induced a decrease in bone marrow cellularity and increased extramedullary hematopoiesis in the spleen (Johns et al., 2009; MacNamara et al., 2009). In our model, the HSPC frequency at 21 dpi in the spleen was around eighteen-fold higher than that in controls. It is plausible to propose that extramedullary hematopoiesis following infection contributes to hematological maintenance. Previously, we demonstrated that leukocyte numbers were enhanced around three times in response to infection at 21 and 28 dpi (Antunes et al., 2019).

Albeit the bone marrow seems to maintain hematopoietic function expanding HSPCs, the evident unbalance between non-committed and committed progenitor cell number remains along with the acute infection. Along with the changes in the bone marrow, the thymus was also targeted, with a reduction in the numbers of differentiating thymocytes, particularly on CD4^+^CD8^+^ cells. Conceptually these findings clearly show that both types of primary lymphoid organs were compromised. Moreover, the enhancement of LSK-like cells and progressive expansion of HSPCs triggered in the spleen highlight the hematopoietic disturbance following oral acute *T. cruzi* infection.

Overall, regarding the mechanisms involved in HSPC activation causing effects in differentiation or mobilization, as well as the direct impact in hematological changes, immunosuppression by LSK-like cells as a mechanism in parasite persistence and immune cell replenishment remain to be explored during the acute phase of oral *T. cruzi* infection.

## Limitations of the Study

Although our study unequivocally shows that *T. cruzi* infection impacts hematopoiesis, a more detailed characterization of the hematopoietic tree and the peripheral correlates was not approached. Also, analyses of the infected cellular components of the bone marrow and its consequence upon the hematopoietic mediators and hematopoietic hierarchy, as well as the investigation of hematological changes after 21 dpi, still need to be done.

## Data Availability Statement

The raw data supporting the conclusions of this article will be made available by the authors, without undue reservation.

## Ethics Statement

The animal study was reviewed and approved by Institutional Ethics Committee for Animal Research of the Oswaldo Cruz Foundation (CEUA-Fiocruz, License: L-028/2016).

## Author Contributions

JM coordinated the study, conceived the experiments, analyzed the data, and participated in writing the manuscript. AM-S contributed to the conception and design of the study, performed the experiments of parasite infection and flow cytometry, and also contributed to the writing of the manuscript. JA-S did most of the histopathological analyses related to the bone marrow and contributed to the writing of the manuscript. DJMA performed the experiments. CCM provided the parasites for infecting mice and contributed to the writing of the manuscript. AB, WS, and DFdO contributed to the conception and design of the study and in writing the manuscript. MP-M contributed to the histopathological analysis and in writing the manuscript. DA and AZ discussed the content and contributed to the manuscript revision. All authors reviewed and/or edited the manuscript prior to submission.

## Funding

This work was supported by the Coordination for the Improvement of Higher Education Personnel (CAPES) and the Brazilian National Research Council (CNPq; grant #142250/2016-3 and INCT-NIM #465489/2014-1), Carlos Chagas Foundation for funding research in the State of Rio de Janeiro (FAPERJ, grant #250332), the Oswaldo Cruz Institute/Fiocruz (intramural funding), and Mercosur Fund for Structural Convergence (FOCEM, grant #003/2011). The funders had no role in study design, data collection and analysis, decision to publish, or preparation of the manuscript.

## Conflict of Interest

The authors declare that the research was conducted in the absence of any commercial or financial relationships that could be construed as a potential conflict of interest.

## Publisher’s Note

All claims expressed in this article are solely those of the authors and do not necessarily represent those of their affiliated organizations, or those of the publisher, the editors and the reviewers. Any product that may be evaluated in this article, or claim that may be made by its manufacturer, is not guaranteed or endorsed by the publisher.

## Supplementary Material

Supplementary Figure 1 Flow cytometry gating strategy for analyzing bone marrow progenitors. The gate strategy was defined by backgating Lin^-^ cells within FSC vs SSC dot plot. Follow this setting, the graph was ungated and all analyses were performed in singlets. The positive expression of progenitor cell markers was based on FMO controls to check for fluorescence spread and isotype controls to determine the level of non-specific binding.

Supplementary Figure 2 Parasitemia quantification. Male BALB/c mice were infected through the oral cavity with 5× 10^4^ insect-derived metacyclic forms of *T. cruzi*. Parasitemia (mean and SEM) was assessed during the acute phase. Parasitemia was calculated by the Pizzi-Brener method. The total number of animals in each time point was obtained from different experiments. n: 7 dpi =21, 10 and 14 dpi = 15, 18 dpi = 18, 21 dpi = 11, 23 dpi = 6, 28 dpi = 8, 36, 40, 42, 46, 48 and 53 = 4.

Supplementary Figure 3 immunofluorescence of bone marrow following orally induced *T. cruzi* acute infection. **(A-C and D-F)** represent respectively immunofluorescence for *T. cruzi* detection of femur sections of infected mice at 14 dpi and 21 dpi. Representative micrographs derive from experiment with 5 mice at 14 and 21 dpi. The tissue was labeled with primary antibody with reactivity to *T. cruzi* and secondary antibody anti-mouse Alexa Fluor 488, green; eosinophil autofluorescence, red; and nucleus, DAPI, white. Bars show the magnification of each micrograph.

Supplementary Figure 4 Splenic evaluation throughout acute phase of *T. cruzi* infection. **(A)** Spleen size measurements were made in all tissue sections in the control and infected mice at 7, 14 and 21 dpi. Panels **(B, C)** depict panoramic view and a higher magnification of the red pulp area showing exacerbated red pulp at 14 dpi. Panels **(D, E)** reveal similar areas at 21 dpi mice. Apparent megakaryocyte hyperplasia (*), intense erythropoiesis **(E)** and incipient myelopoiesis foci (M) were signposted. Tissue sections were stained by hematoxylin and eosin. The data are representative of 5 mice per group, both at 14 and 21 dpi. Bars show the magnification of each micrograph in each panel. The data are representatives of an experiment with 8 animals from control and 5, 3 and 5 animals from infected mice, at 7, 14 and 21 dpi, respectively. Statistical differences were ascertained with the Two-way Anova test with p<0.0001.
