# Supplementary figures and images for: Oral Trypanosoma cruzi Acute Infection in Mice Targets Primary Lymphoid Organs and Triggers Extramedullary Hematopoiesis
Source: Front Cell Infect Microbiol. 2022 Mar 24;12:800395. doi: 10.3389/fcimb.2022.800395 (PMC8990980; doi:10.3389/fcimb.2022.800395)

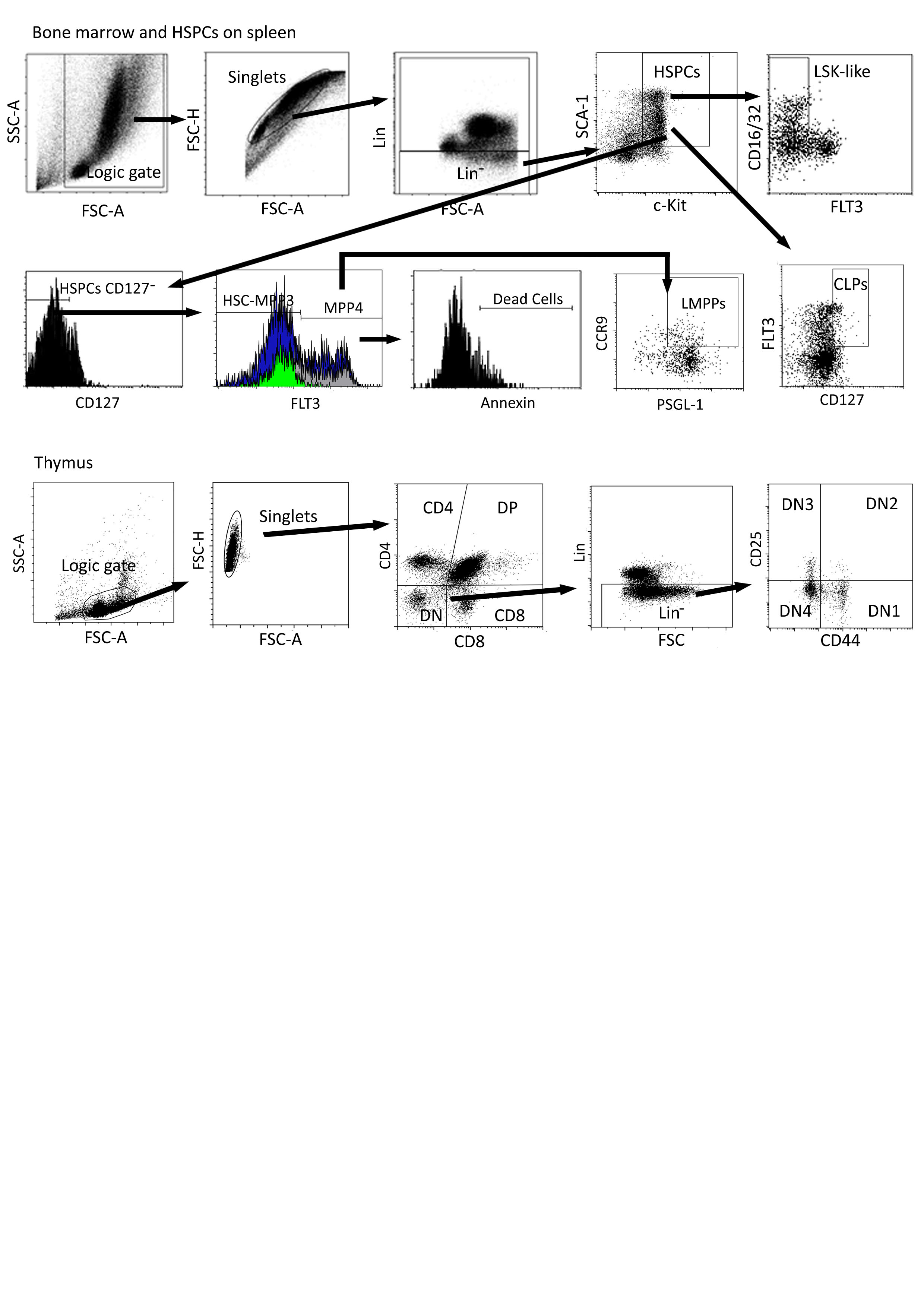

Supplement: Supplementary Figure 1 — Flow cytometry gating strategy for analyzing bone marrow progenitors. The gate strategy was defined by backgating Lin- cells within FSC vs SSC dot plot. Follow this setting, the graph was ungated and all analyses were performed in singlets. The positive expression of progenitor cell markers was based on FMO controls to check for fluorescence spread and isotype controls to determine the level of non-specific binding. [file Image_1.jpeg]

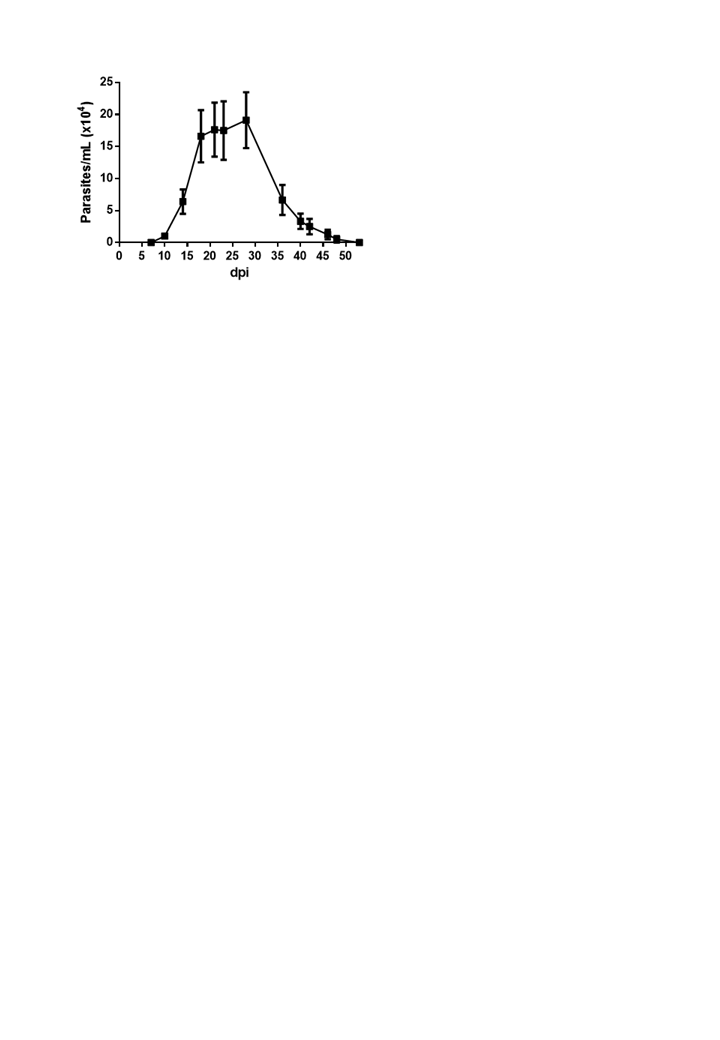

Supplement: Supplementary Figure 2 — Parasitemia quantification. Male BALB/c mice were infected through the oral cavity with 5× 10^4^ insect-derived metacyclic forms of T. cruzi. Parasitemia (mean and SEM) was assessed during the acute phase. Parasitemia was calculated by the Pizzi-Brener method. The total number of animals in each time point was obtained from different experiments. n: 7 dpi =21, 10 and 14 dpi = 15, 18 dpi = 18, 21 dpi = 11, 23 dpi = 6, 28 dpi = 8, 36, 40, 42, 46, 48 and 53 = 4. [file Image_2.tif]

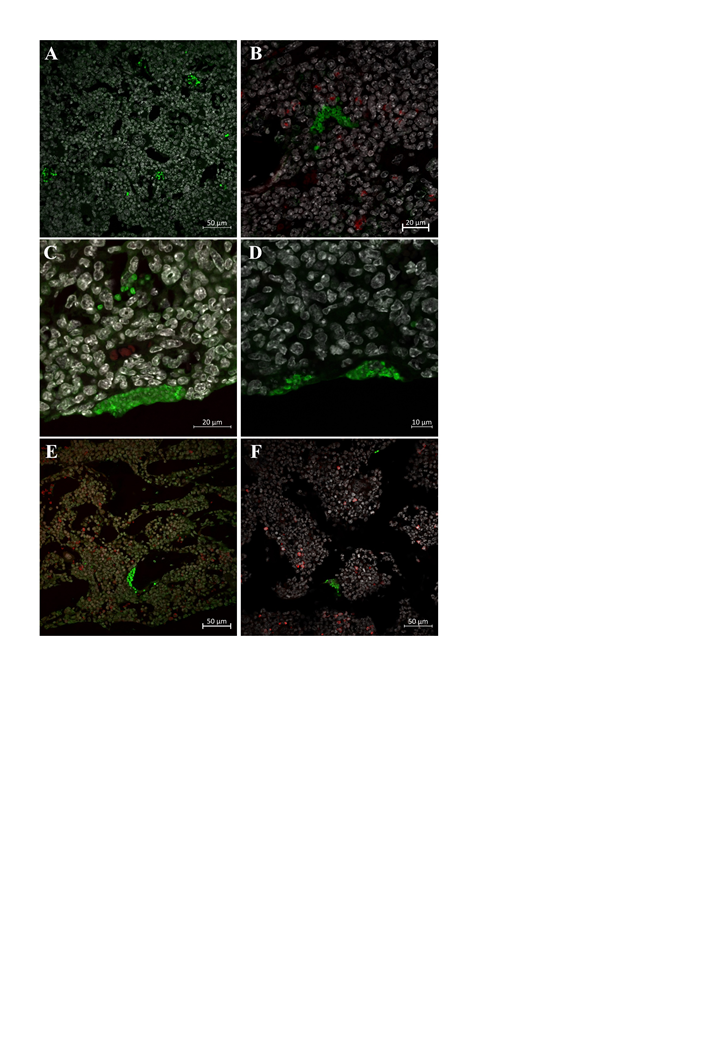

Supplement: Supplementary Figure 3 — immunofluorescence of bone marrow following orally induced T. cruzi acute infection. (A-C and D-F) represent respectively immunofluorescence for T. cruzi detection of femur sections of infected mice at 14 dpi and 21 dpi. Representative micrographs derive from experiment with 5 mice at 14 and 21 dpi. The tissue was labeled with primary antibody with reactivity to T. cruzi and secondary antibody anti-mouse Alexa Fluor 488, green; eosinophil autofluorescence, red; and nucleus, DAPI, white. Bars show the magnification of each micrograph. [file Image_3.tif]

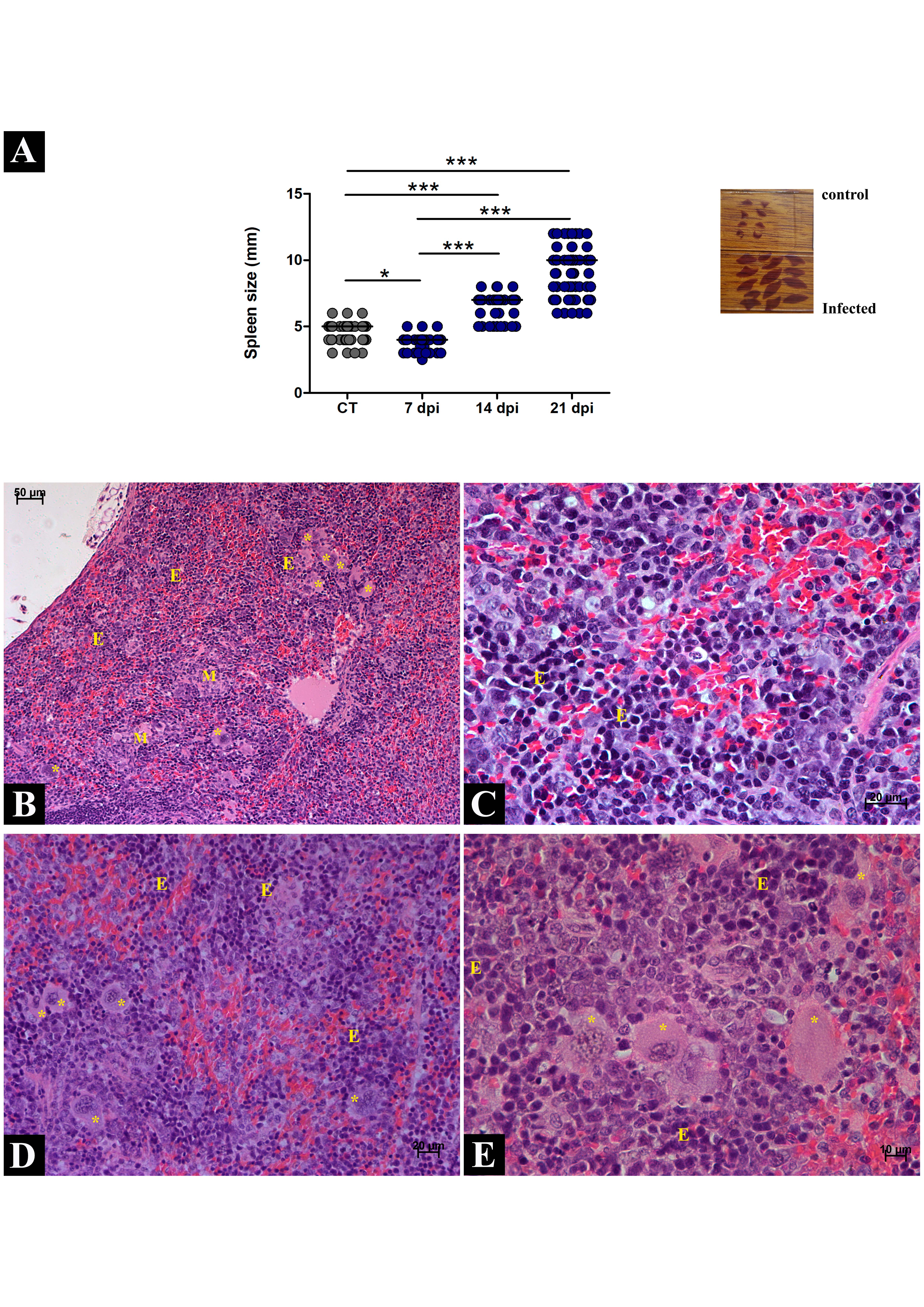

Supplement: Supplementary Figure 4 — Splenic evaluation throughout acute phase of T. cruzi infection. (A) Spleen size measurements were made in all tissue sections in the control and infected mice at 7, 14 and 21 dpi. Panels (B, C) depict panoramic view and a higher magnification of the red pulp area showing exacerbated red pulp at 14 dpi. Panels (D, E) reveal similar areas at 21 dpi mice. Apparent megakaryocyte hyperplasia (*), intense erythropoiesis (E) and incipient myelopoiesis foci (M) were signposted. Tissue sections were stained by hematoxylin and eosin. The data are representative of 5 mice per group, both at 14 and 21 dpi. Bars show the magnification of each micrograph in each panel. The data are representatives of an experiment with 8 animals from control and 5, 3 and 5 animals from infected mice, at 7, 14 and 21 dpi, respectively. Statistical differences were ascertained with the Two-way Anova test with p<0.0001. [file Image_4.jpeg]

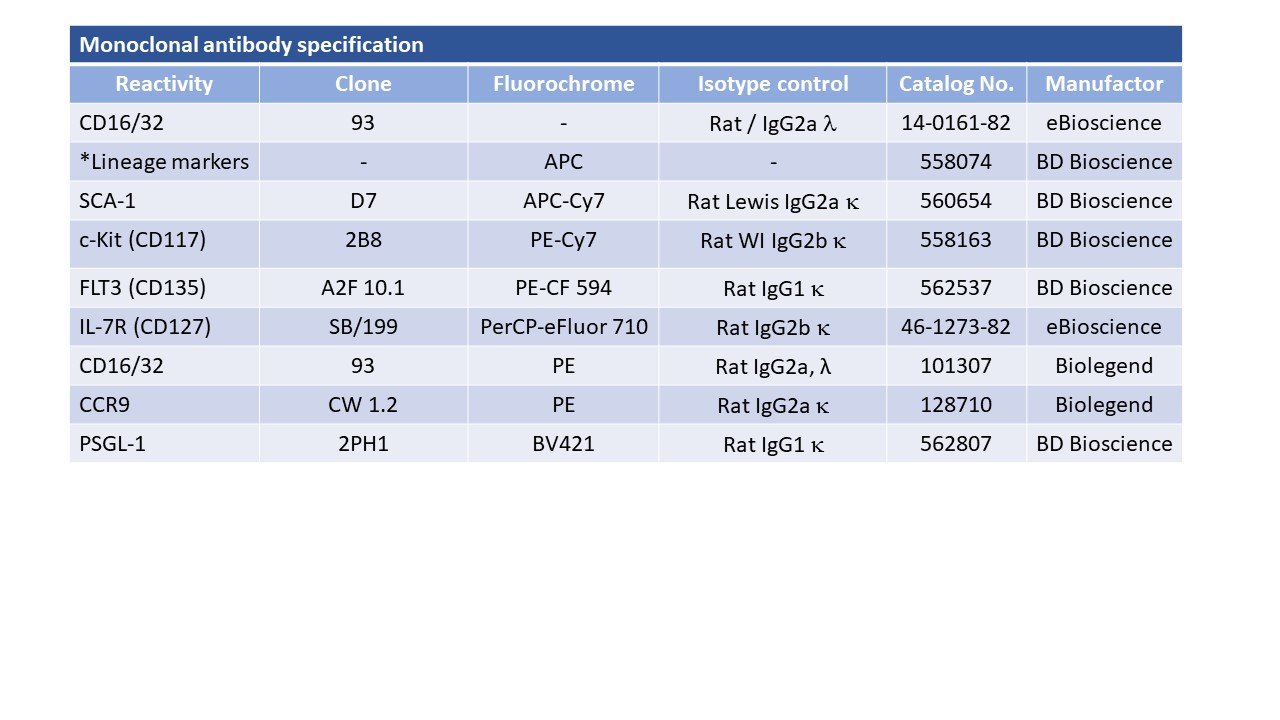

Supplement: Supplementary file 5 [file Image_5.jpeg]
